# Clinical impact of progesterone levels on HCG trigger day in a follicular long-term IVF protocol

**DOI:** 10.3389/fendo.2025.1593079

**Published:** 2025-11-20

**Authors:** Xiao Han, Na Song, Ya Zhang, Jingjing Wang, Fang Zhao, Yanping Zhang, Zhanrong Shi, Yuzhen Lv, Menglan Wu, Fang Yuan, Guangli Zhu

**Affiliations:** Reproductive Medicine Center, Jiaozuo Women’s and Children’s Hospital, Jiaozuo, China

**Keywords:** progesterone, follicular long-term protocol, HCG trigger day, fresh embryo transfer, clinical pregnancy

## Abstract

**Background:**

Previous studies have shown that elevated progesterone (P4) levels on human chorionic gonadotropin (HCG) trigger days may affect endometrial tolerance. However, no standardized threshold has been established for the impact of late follicular phase P4 on clinical outcomes. This study aimed to assess the influence of P4 levels on the day of HCG trigger in a follicular long-term protocol on clinical outcome.

**Methods:**

This retrospective study analyzed 889 fresh *in vitro* fertilization-embryo transfer cycles, each involving a single top-quality cleavage-stage embryo (CSE) or blastocyst transfer. Univariate and multivariate logistic regression analyses, threshold effect analysis, and curve fitting were carried out.

**Results:**

A significant correlation was identified between the P4 level and both the clinical pregnancy rate ([CPR] OR: 0.54, 95% CI: 0.37 – 0.81, *p* = 0.003) as well as the ongoing pregnancy rate ([OPR] OR: 0.68, 95% CI: 0.46 – 0.99, *p* = 0.047) in blastocyst transfer. However, no significant relationship was found between P4 level and CPR or OPRs in CSE transfer. The OPR displayed a linear relationship with the P4 level in CSEs. In blastocyst transfer, the CPR followed a parabolic pattern, initially declining gradually at higher P4 levels and then dropping sharply when P4 reached ≥ 1.0 ng/mL. On the other hand, the OPR displayed a reverse U-shaped curve, increasing with P4 levels up to 1.0 ng/mL before declining at higher levels. The OPR significantly declined when the P4 level exceeded 1.0 ng/mL.

**Conclusions:**

In blastocyst transfer cycles, the P4 level on the HCG trigger day shows a curvilinear association with the outcomes of pregnancy. The optimal threshold was found to be as 1.0 ng/mL.

## 1 Introduction

Under normal physiological conditions, progesterone (P4) levels rise following ovulation, facilitating the endometrium to move from a proliferative to a secretory phase, thus supporting embryo implantation. However, in assisted reproductive technology (ART), factors such as gonadotropin (Gn) use and the development of multiple follicles during controlled ovarian hyperstimulation may lead to early increases in P4 levels. Along with endometrial receptivity (1, 2), these early increases might compromise oocyte and embryo quality (3, 4). A meta-analysis of > 60,000 cycles found that elevated levels of P4 on the HCG trigger day were linked to lower pregnancy rates in fresh transfer cycles involving ovarian stimulation with GnRH agonists and Gn (5). Zhang (6) et al. reported that increased P4 levels in the late follicular phase negatively affected clinical pregnancy (CP) outcomes in pituitary down-regulation treatment cycles. However, other studies have found no significant association between elevated late follicular phase P4 and pregnancy outcomes (7–9). Moreover, no standardized threshold has been established for the impact of late follicular phase P4 on clinical outcomes. Variability in patient age, ovarian response, ovulation induction protocols, embryo transfer number, and embryo quality across studies may account for these differing conclusions. Several studies indicate that after cleavage-stage embryo (CSE) transfer, a high P4 serum level significantly affects clinical outcomes (1, 10), whereas findings related to blastocyst transfer remain inconsistent (11, 12). Limited research has explored how elevated P4 levels impact pregnancy outcomes across different embryo types. The present study aimed to examine the effects of increased P4 levels on pregnancy in various embryo transfer types on HCG days.

## 2 Materials and methods

### 2.1 Study population

This study was a retrospective, single-center cohort analysis. It included patients who had undergone *in vitro* fertilization (IVF) or intracytoplasmic sperm injection (ICSI) treatment. Eligibility criteria required controlled ovarian stimulation using the follicular long-term protocol and the transfer of a single top-quality CSE or blastocyst in a fresh cycle between December 2016 and July 2024 at the Center for Reproductive Medicine, Jiaozuo Women’s and Children’s Hospital.

Exclusion criteria included chromosomal abnormalities in either partner, uterine malformations (such as arcuate uterus, unicornuate uterus, rudimentary uterine horn, and septate uterus), and intrauterine conditions that could affect embryo transfer (including uterine cavity adhesion, endometrial polyps, hydrosalpinx with reflux into the uterine cavity or a history of endometrial tuberculosis). Furthermore, individuals diagnosed with recurrent pregnancy loss, endometritis, or endometrial hyperplasia were excluded from the study. As per the center’s policy, patients with HCG day P4 levels exceeding 2.5 ng/mL underwent whole embryo freezing; thus, only those with HCG day P4 levels ≤ 2.5 ng/mL were included. After applying these criteria, the study analyzed 889 eligible fresh transfer cycles. The study was approved by the hospital’s ethics committee and institutional review board (approval no. 2024-KY-LHGJ20240702).

### 2.2 Ovarian stimulation

Gn-releasing hormone agonist (GnRH-a) therapy was initiated with leuprolide acetate (3.75 mg/dose; Shanghai Lizhu Pharmaceutical Co., Ltd.) on days 2–3 of menstruation. After 30 days, follicle-stimulating hormone (FSH), estradiol (E2), luteinizing hormone (LH), and P4 levels were assessed. When the hormone levels reached the specified thresholds, ovarian stimulation was initiated using recombinant human FSH (Gonadaphen, Merck Serono). The dosage was carefully adjusted based on multiple factors, including the patient’s age, body weight, and antral follicle count (AFC), to optimize the response to treatment. When follicles measuring ≥ 18 mm in diameter constituted at least 40% of the total follicles, Gn administration was discontinued. Ovulation was then induced with an intramuscular injection of 2,000 IU chorionic Gn and a percutaneous injection of 250 μg recombinant chorionic Gn on the same day. Oocyte retrieval was performed 37.5 hours post-trigger using ultrasound-guided aspiration.

### 2.3 P4 measurement

Blood samples were collected on the day of HCG injection, specifically between 7:00 a.m. and 9:00 a.m., to ensure consistency in sample collection timing. P4 levels were analyzed using electrochemiluminescence on a Roche Cobas 6000 system. Routine internal quality control was maintained, and calibration was performed as needed, particularly when results fell outside the normal range or when a new reagent batch was introduced.

### 2.4 Scoring of embryos

Prior to performing the transfer of embryo transfer, individual embryos were subjected to evaluation based on their growth rate, fragmentation level, and the uniformity of their cells. Those containing 7 to 9 blastomeres, well-defined morphology, a homogeneous cytoplasm, and a rate of fragmentation below 10% were categorized as top-quality. For blastocyst assessment, the Gardner scoring system was applied, considering embryos rated 3BB or higher as top-quality.

### 2.5 Outcome measurement

CP was determined by identifying a gestational sac using an ultrasound examination, which was conducted between 4 and 6 weeks after the embryo transfer procedure. To classify an ongoing pregnancy (OP), it must progress for at least 12 weeks, with ultrasound confirmation of a viable fetus and detectable cardiac activity.


$$CP\;rate\;(\%)=\frac{\text{No}.\text{ of CP cycles}}{\text{number of transfer cycles}}*100$$

$$OP\;rate(\%)=\frac{\text{No}.\text{ of OP cycles}}{\text{number of transfer cycles}}*100$$

### 2.6 Statistical analysis

Statistical analyses were employed using SPSS software (version 22.0) and R program (version 4.4.1). The Shapiro–Wilks test was applied to identify the distribution of the data. For continuous variables, those following a non-normal distribution were expressed as the median with interquartile ranges (25^th^ – 75th percentile), while data following a normal distribution were presented as the mean ± standard deviation (SD)and analyzed using the Student’s t-test to compare groups. The Mann–Whitney U test was carried out to compare differences in median values for non-normally distributed variables. Counts and percentages were used to summarize categorical variables, and groups were compared by using the Pearson’s Chi-square test.

To further analyze the data, binary logistic regression, curve fitting, and threshold effect analysis were conducted across all cycles. The selection of confounding variables was based on their potential relevance to the outcomes or their influence on the effect estimate, defined as a change of at least 5%. Smooth curve fitting techniques were applied to investigate possible non-linear associations between P4 levels and pregnancy outcomes. Furthermore, piece-wise linear regression analysis was performed for the assessment of the threshold effect of P4 levels on pregnancy outcomes. Statistical significance was defined as a *p*-value of less than 0.05.

## 3 Results

### 3.1 General characteristics of patients

This research comprised a total of 889 cycles. Baseline characteristics assessed included women’s age, BMI, basal E2, FSH, and P4 levels, infertility type and duration, type of fertilization, AFC, Gn dosage and duration, E2 and LH levels, P4 level on the HCG trigger day (HCG-T day), endometrial thickness, the number of oocytes retrieved, embryo type, and the number of two-pronuclear embryos. The overall CP rate (CPR) per embryo transfer cycle was 64.53% (573/889), while the OP rate (OPR) per embryo transfer cycle was 55.74% (495/889). **Table 1** summarizes the demographic and IVF/ICSI characteristics of the patients.

**Table 1:** Characteristics of patients undergoing fresh cycles with one top quality embryo transfer.

Item	Mean (SD)/median (Q1-Q3)	N (%)
Female age	30 (28-33)	
BMI	23.5 (21.00-26.70)	
Duration of infertility (year)	3.25 (1.75-5.00)	
Fertilization type		
IVF		713 (80.29)
ICSI, rescue-ICSI		136 (15.32)
IVF & rescue-ICSI		39 (4.39)
Infertility type		
Primary Infertility		450 (50.68)
Secondary Infertility		438 (49.32)
Basal FSH concentration (mIU/ml)	6.24 (5.37-7.21)	
Basal E2 concentration (pg/ml)	36.51 (28.75-48.15)	
Basal P4 concentration (ng/ml)	0.27 (0.17-0.40)	
AFC	20 (15-24)	
Gn dosage (IU)	2850.00 (2090.63-3750.00)	
Gn duration (day)	12.00 (11.00-13.00)	
E2 concentration on hCG trigger day (pg/ml)	3588.50 (2676.00-4783.75)	
LH concentration on hCG trigger day (mIU/ml)	1.10 (0.81-1.47)	
P4 concentration on hCG trigger day (ng/ml)	0.71 (0.44-1.03)	
EMT on hCG trigger day (mm)	10.80 (9.30-12.88)	
No. of retrieved oocytes	13.00 (10.00-16.00)	
No. of 2PN embryo	10.00 (7.00-12.00)	
Embryo type		
cleavage-stage embryo		350 (39.41)
blastocyst		538 (60.59)
Clinical pregnancy in the fresh cycle (;%)		573 (64.53)
Ongoing pregnancy in the fresh cycle (;%)		495 (55.74)
number of transferable embryos	6.00 (4.00-8.00)	
high-quality embryo number	5.00 (3.00-7.00)	

BMI, body mass index; AFC, antral follicle counting; Gn, gonadotropin; E2, estradiol; LH, luteinizing hormone; P4, progesterone; EMT, endometrium thickness.

Patients with total fertilization failure in IVF were assigned to the Rescue-ICSI group, those with low fertilization rate in IVF were allocated to the IVF & Rescue-ICSI group.

### 3.2 Univariate logistic regression analysis

Univariate analysis was conducted to investigate factors influencing clinical pregnancy and ongoing pregnancy outcomes following the transfer of a single top-quality embryo. The age of a female was found to negatively affect both CPR (p=0.006) and OPR (p<0.001). The count of oocytes retrieved (p=0.017), and AFC (p<0.001) were found associated with a higher CPR. Furthermore, the duration of infertility (p=0.047), AFC (p=0.001), and the number of oocytes retrieved (p=0.002) were linked to an increased OPR. In cases where blastocyst transfer was performed, both CPR (p<0.001) and OPR (p<0.001) were found to be significantly elevated. This suggests that blastocyst transfer may be associated with improved pregnancy outcomes. Comprehensive data supporting these findings can be found in **Table 2**, which provides a detailed breakdown of the results.

**Table 2:** Univariate analysis of factors associated with pregnancy outcomes.

Item	Clinical pregnancy		Ongoing pregnancy	
	OR (95%CI)	P	OR (95%CI)	P
Female age	0.95 (0.92-0.99)	0.006	0.93 (0.90-0.96)	<0.001
BMI	1.03 (0.99-1.06)	0.176	0.99 (0.96-1.03)	0.702
Duration of infertility (year)	0.97 (0.92-1.02)	0.204	0.95 (0.90-1.00)	0.047
Fertilization type				
IVF	1		1	
ICSI, rescue-ICSI	0.71 (0.488-1.03)	0.070	0.83 (0.58-1.20)	0.320
IVF & rescue-ICSI	1.34 (0.66-2.74)	0.421	1.40 (0.71-2.73)	0.328
Infertility type				
Primary Infertility	1		1	
Secondary Infertility	0.86 (0.65-1.13)	0.285	0.80 (0.61-1.04)	0.101
Basal FSH (mIU/ml)	0.93 (0.85-1.02)	0.104	0.94 (0.86-1.03)	0.158
Basal E2 (pg/ml)	1.00 (0.99-1.01)	0.887	1.00 (0.99-1.00)	0.496
Basal P4 (ng/ml)	0.78 (0.51-1.17)	0.229	0.82 (0.54-1.23)	0.329
AFC	1.05 (1.03-1.08)	<0.001	1.04 (1.02-1.07)	0.001
Gn dosage (IU)	1.00 (1.00-1.00)	0.276	1.00 (1.00-1.00)	0.088
Gn duration (day)	0.99 (0.92-1.07)	0.752	0.99 (0.92-1.06)	0.725
E2 level on hCG trigger day (pg/ml)	1.00 (1.00-1.00)	0.541	1.00 (1.00-1.00)	0.075
LH level on hCG trigger day (mIU/ml)	1.05 (0.85-1.30)	0.651	0.99 (0.81-1.22)	0.950
P4 level on hCG trigger day (ng/ml)	0.79 (0.59-1.05)	0.104	0.88 (0.67-1.16)	0.367
EMT on hCG trigger day (mm)	1.00 (0.95-1.06)	0.999	1.01 (0.96-1.06)	0.787
No. of retriev1.04 (1.01-1.06)	1.04 (1.01-1.06)	0.017	1.04 (1.02-1.07)	0.002
No. of 2PN embryo	1.07 (1.04-1.11)	<0.001	1.08 (1.04-1.11)	<0.001
Embryo type				
cleavage-stage embryo	1		1	
blastocyst	2.67 (2.01-3.55)	<0.001	2.38 (1.81-3.14)	<0.001

confidence interval (CI); odds ratio (OR); body mass index (BMI); antral follicle counting (AFC); gonadotropin (Gn); estradiol(E2), luteinizing hormone (LH), progesterone(P4); endometrium thickness (EMT).

### 3.3 Multivariate logistic regression analysis

Age, BMI, and infertility duration were among the confounding factors that were controlled for using multivariate logistic regression analysis. The analysis displayed a significant correlation between the P4 level and the CPR post-blastocyst transfer (OR: 0.54, 95% CI: 0.37 - 0.81, *p* = 0.003). A significant correlation was seen between P4 levels and continued pregnancy rates in blastocyst transfer (OR: 0.68, 95% CI: 0.46 - 0.99, *p* = 0.047) (**Table 3**).

**Table 3:** Associations between P4 and clinical outcomes of fresh cycles using multivariable logistic regression analysis.

Outcome	Clinical pregnancy		Ongoing pregnancy	
	OR (95%CI)	P	OR (95%CI)	P
cleavage-stage embryo	1.10 (0.70-1.74)	0.670	1.18 (0.75-1.87)	0.475
blastocyst	0.54 (0.37-0.81)	0.003	0.68 (0.46-0.99)	0.047
total	0.83 (0.61-1.13)	0.236	0.87 (0.64-1.18)	0.378

Adjusted for female age; BMI; duration of infertility; AFC; No. of retrieved oocytes.

CI, confidence interval ; OR, odds ratio.

### 3.4 Curve fitting analysis between P4 level and clinical outcomes across various embryo types

**Figure 1:**
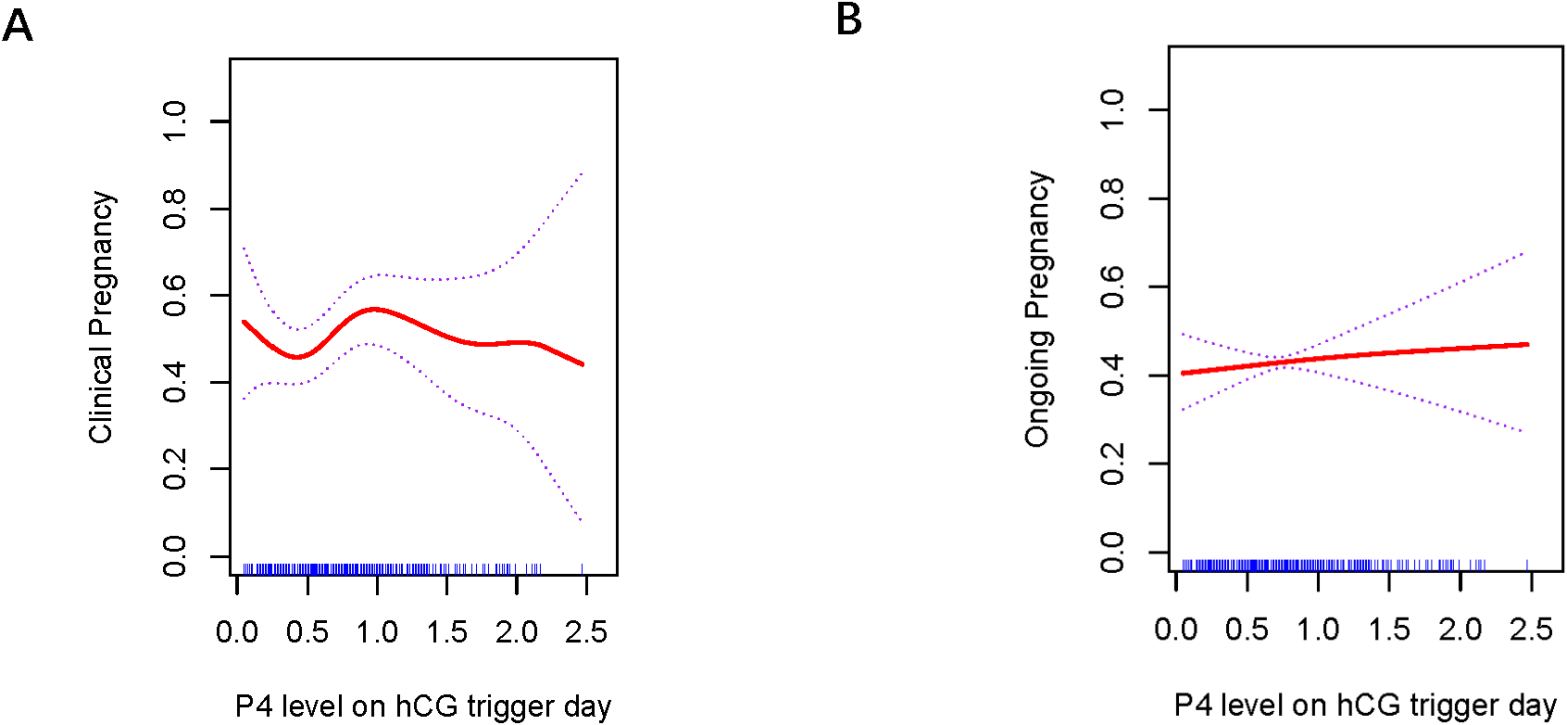
Association between progesterone concentration and clinical outcomes in cleavage-stage embryo transfers **(A)** Clinical Pregnancy, **(B)** Ongoing Pregnancy. The fitted line was adjusted for female age, BMI, antral follicle count, and the number of retrieved oocytes.

**Figure 2:**
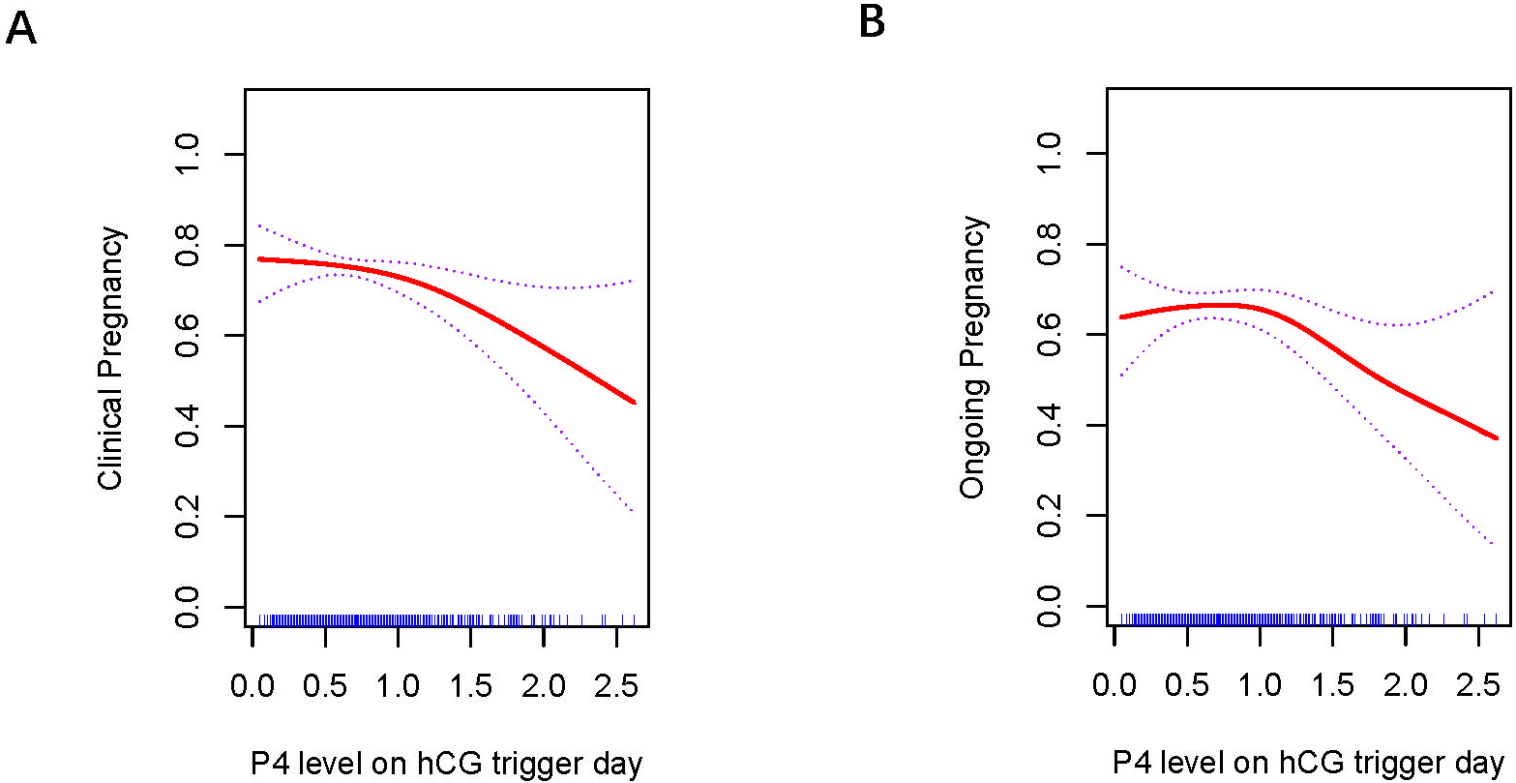
Association between progesterone concentration and clinical outcomes in blastocyst transfers **(A)** Clinical Pregnancy, **(B)** Ongoing Pregnancy. A threshold, nonlinear association between the progesterone concentration and clinical and ongoing pregnancy rate was found in blastocyst transfer in a generalized additive model. The fitted line was adjusted for female age; BMI, duration of infertility, antral follicle count, and the number of retrieved oocytes.

**Figures 1**, **2** present the fitted curves, adjusted for confounding variables, illustrating the correlation between P4 level and clinical outcomes. In the case of CSEs, the CPR demonstrated a nonlinear relationship with P4 levels, whereas the OPR exhibited a linear association with P4 levels. For blastocyst transfer, the CPR followed a parabolic curve, showing a slow decline followed by a rapid decrease at higher P4 levels. The OPR, on the other hand, increasing initially and then decreasing as the P4 level increased.

### 3.5 Threshold effect of P4 on fresh cycle outcomes

Threshold effects were identified for the curves in blastocyst transfer. The analysis of P4 levels and clinical outcomes is summarized in **Table 4**. CPR initially tended to decrease as P4 level increased, but the decline became rapid when P4 reached ≥ 1.0 ng/mL(p=0.008). OPR tended to increase with higher P4 levels, peaked at 1.0 ng/mL, and then declined significantly when P4 > 1.0 ng/mL(p=0.006).

**Table 4:** Threshold effect analysis of progesterone concentration and clinical outcomes in fresh cycles using the piece-wise linear regression method.

Blastocyst	Cutoff of P4 level	Effect size (OR)	95%CI	P
Clinical Pregnancy(CPR)	<1.0	1.14	0.52-2.51	0.740
	≥1.0	0.32	0.13-0.74	0.008
Ongoing Pregnancy (OPR)	<1.0	1.41	0.68-2.94	0.359
	≥1.0	0.31	0.13-0.71	0.006

Adjusted for female age; BMI; AFC; No. of retrieved oocytes; duration of infertility

CI, confidence interval; OR, odds ratio.

## 4 Discussion

The present study demonstrated that elevated serum P4 levels on the HCG-T day negatively impacted pregnancy outcomes, with this effect being more significant in blastocyst transfer. Furthermore, to minimize bias related to embryo quantity and quality, only one top-quality CSE or blastocyst transfer cycle was included in the analysis.

Thresholds for P4 levels on the day of HCG trigger and their impact on clinical outcomes in the transfer cycles of fresh embryos remain controversial. A 2020 retrospective study of over 8,000 cycles found that elevated P4 levels reduce CPR and live birth rates in both blastocyst transfers as well as CSEs, recommended embryo freezing and delayed transfer if P4 levels are ≥ 1.75 ng/mL (13). A study by the Santos group reported that P4 levels > 1.5 ng/mL significantly decreased the live birth rates in patients (14). Another 2022 study indicated that even mildly elevated P4 levels (1.0 – 1.5 ng/mL) affect CPR in single blastocyst transfers by using the follicular long-term protocol in fresh cycles (15). The study by Zhao and colleagues also reported that mildly elevated P4 levels (1.0 – 1.5 ng/mL) on the HCG-T day led to a significantly lower CPR in the antagonist protocol as compared with normal P4 levels, with CPR declining significantly once P4 levels exceeded 1.4 ng/mL (16). Furthermore, a meta-analysis of over 60,000 cycles found an inverse relationship between P4 levels and pregnancy rates when P4 levels reached or exceeded 0.8 ng/mL in the late follicular phase (5). Possible reasons for differences between the results of these studies and the present study include different sample sizes, different ovarian stimulation protocols, different types of embryos included, and different P assays used.

The mechanism through which early elevation of P4 levels impacts clinical outcomes in fresh embryo transfer cycles is still debated, especially concerning oocyte and embryo quality (17). Research has shown that elevated P4 on the HCG-T day significantly decreases CPR, even in patients with high-quality embryos. This suggests that the adverse impacts of premature P4 elevation are not influenced by the embryo transfer stage, embryo quality, the woman’s age, or ovarian response levels (18). An increasing body of research indicates that elevated P4 levels influence gene expression and epigenetic modifications related to immune tolerance in endometrial cells, impairing the implantation window and reducing endometrial receptivity (17, 19). A study examining the endometrial transcriptome during the implantation window found significant differences in gene expression patterns associated with natural killer cell-mediated cytotoxicity pathways. These disparities were seen in the endometrium of individuals displaying increased blood P4 levels on the HCG-T day, in contrast to those with standard endometrial expression patterns (20). Jiang et al. indicated that increased blood P4 levels on the HCG-T day did not influence CPRs in CSE transfers. In IVF-ET cycles using a short-acting GnRHa downregulation technique, elevated P4 levels correlated with reduced CPRs for blastocyst transfers (21). Similarly, Kong et al. discovered that increased serum P4 levels on the HCG-T day did not affect the CPR of CSE transfers but were associated with a decreased CPR for blastocyst transfers in IVF-ET cycles using a long-term follicular protocol (22). Bo et al. (9) reported that P4 levels on the HCG-T day had no effect on the CPR or live birth rates in CSE transfers. These findings align with the present study, supporting the hypothesis that the differential effect may be due to the timing of blastocyst and CSE transfers. Blastocyst transfer, typically carried out on day 5 after oocyte retrieval, may be affected by early endometrial transformation, leading to premature closing of the implantation window and potential implantation failure. In contrast, CSE transfers occur on day 3, making them more likely to coincide with the optimal endometrial implantation window.

Our study revealed that P4 level on HCG-T day differentially impact clinical outcomes between cleavage-stage embryo and blastocyst transfers, with blastocyst transfers demonstrating greater susceptibility to P4 elevation. Furthermore, we established a clinically significant threshold for P4 level on HCG-T day in blastocyst transfer cycles, which may help guide clinical transfer strategies and reduce unnecessary whole embryo freezing. However, there are some limitations of the study. The retrospective nature of this study can lead to some degree of bias, despite the implementation of strict inclusion and exclusion criteria. Moreover, as this study focused solely on cycles using a follicular long-term protocol, the findings may not be generalizable to other protocols. Further validation through high-quality randomized controlled trials is necessary.

## 5 Conclusion

Blastocysts show a higher sensitivity to variations in serum P4 levels compared to CSEs. In blastocyst transfer cycles, when the P4 level on the HCG-T day was <1.0 ng/ml, the CPR tended to decrease and the OPR tended to increase with increasing P4 levels, and when the P4 level was ≥1.0 ng/ml, the CPR and the OPR decreased significantly with increasing P4 levels, and the optimal threshold for the P4 level on the HCG-T day was 1.0 ng/ml.

## Data Availability

The raw data supporting the conclusions of this article will be made available by the authors, without undue reservation.
